# Patient-centered boundary mechanisms to foster intercultural partnerships in health care: a case study in Guatemala

**DOI:** 10.1186/s13002-017-0170-y

**Published:** 2017-08-08

**Authors:** Martin Hitziger, Mónica Berger Gonzalez, Eduardo Gharzouzi, Daniela Ochaíta Santizo, Regina Solis Miranda, Andrea Isabel Aguilar Ferro, Ana Vides-Porras, Michael Heinrich, Peter Edwards, Pius Krütli

**Affiliations:** 1 Section of Epidemiology, University of Zurich, Winterthurerstrasse 270, 8057 Zurich, Switzerland; 2 Swiss TPH, Epidemiology and Public Health, Socinstrasse 57, 4057 Basel, Switzerland; 3 Surgical Oncologist, Head of Education and Research, Instituto de Cancerología, 6a Avenida 6-58 Zona 11, 01011 Guatemala, Guatemala; 4 Universidad del Valle de Guatemala, 18 Avenida 11-95, Zona 15,V.H. III, Guatemala, Guatemala; 5 Universidad del Valle de Guatemala, 10 Avenida 7-62, Zona 1, Guatemala, Guatemala; 6 Department of Anthropology, University of Wyoming, 25 Calle 13-55 Zona 16. Ensenada de San Isidro Casa 19D, Guatemala, Guatemala; 7 Research Cluster Biodiversity and Medicines/Centre for Pharmacognosy and Phytotherapy, UCL School of Pharmacy, London, WC1N 1AX UK; 8 Singapore-ETH Centre, 1 CREATE Way, #06-01 CREATE Tower, Singapore, 138602 Singapore; 9 ETH Zurich, TdLab, Universitätsstrasse 22, CHN, 8092 Zurich, Switzerland

**Keywords:** Boundary management, Partnerships, Intercultural health, Integrative medicine, Traditional medicine, Mayan medicine, Central America, Guatemala

## Abstract

**Background:**

Up to one half of the population in Africa, Asia and Latin America has little access to high-quality biomedical services and relies on traditional health systems. Medical pluralism is thus in many developing countries the rule rather than the exception, which is why the World Health Organization is calling for intercultural partnerships to improve health care in these regions. They are, however, challenging due to disparate knowledge systems and lack of trust that hamper understanding and collaboration. We developed a collaborative, patient-centered boundary mechanism to overcome these challenges and to foster intercultural partnerships in health care. To assess its impact on the quality of intercultural patient care in a medically pluralistic developing country, we conducted and evaluated a case study.

**Methods:**

The case study took place in Guatemala, since previous efforts to initiate intercultural medical partnerships in this country were hampered by intense historical and societal conflicts. It was designed by a team from ETH Zurich’s Transdisciplinarity Lab, the National Cancer Institute of Guatemala, two traditional Councils of Elders and 25 Mayan healers from the Kaqchikel and Q’eqchi’ linguistic groups. It was implemented from January 2014 to July 2015. Scientists and traditional political authorities collaborated to facilitate workshops, comparative diagnoses and patient referrals, which were conducted jointly by biomedical and traditional practitioners. The traditional medical practices were thoroughly documented, as were the health-seeking pathways of patients, and the overall impact was evaluated.

**Results:**

The boundary mechanism was successful in discerning barriers of access for indigenous patients in the biomedical health system, and in building trust between doctors and healers. Learning outcomes included a reduction of stereotypical attitudes towards traditional healers, improved biomedical procedures due to enhanced self-reflection of doctors, and improved traditional health care due to refined diagnoses and adapted treatment strategies. In individual cases, the beneficial effects of traditional treatments were remarkable, and the doctors continued to collaborate with healers after the study was completed. Comparison of the two linguistic groups illustrated that the outcomes are highly context-dependent.

**Conclusions:**

If well adapted to local context, patient-centered boundary mechanisms can enable intercultural partnerships by creating access, building trust and fostering mutual learning, even in circumstances as complex as those in Guatemala. Creating multilateral patient-centered boundary mechanisms is thus a promising approach to improve health care in medically pluralistic developing countries.

## Background

### Boundary mechanisms: a novel approach to foster intercultural partnerships in medically pluralistic countries

The World Health Organization (WHO) estimates that one-third of the world’s population, and as much as half the population in some parts of Africa, Asia and Latin America, have no regular access to biomedicine [1]. Indeed, in many developing countries little has changed since Lee [2] noted that biomedicine, although dominant in terms of power, prestige and wealth, was functionally weak in terms of equitable access and widespread utilization. Consequently, medical pluralism – meaning the coexistence and parallel use of traditional, alternative or complementary systems of health care – is the rule rather than the exception in many countries [3, 4]. In 1978, the World Health Organization called for intercultural health teams to provide locally adapted primary health care [5]. Ever since, medical partnerships have been promoted for reasons that include traditional medicine’s accessibility, the credibility and cultural significance it enjoys in the eyes of local people, and in many cases also its clinical effectiveness [6–10]. Some countries have successfully implemented various models to advance intercultural care [3, 10], but many traditional health systems remain neglected, poorly institutionalized or even suppressed.

Many researchers emphasise barriers for intercultural medical collaboration due to (1) inadequate access, (2) absence of trust and (3) lack of mutual understanding linked to disparate knowledge systems.

1. Barriers to access are understood as geographical, economical, organizational or cultural factors hampering patients’ to get the medical services they need [10].
2. Trust is a precondition for collaboration and for the transformations of social relations [11]. Trust is granted due to expectations of interests, moral commitments or psychological dispositions [12] and draws upon collective narratives that are saturated with power, institutions, and history [13]. Collaborative methods can create trust by (i) building on existing relationships, (ii) using trusted intermediaries, and (iii) providing an environment for repeated interactions in project work [14].
3. Knowledge systems are networks of actors, organizations, and objects that bridge knowledge and know-how with action [15]. All health systems are also knowledge systems, which use different explanatory models to construct different interpretations of the same medical condition. This can lead to conflicting expectations, miscommunication, and ultimately to poor clinical care [16].

Boundary management refers to the boundary between different knowledge systems. It is an aproach to bridge the barriers that often hamper communication and collaboration across these boundaries. Boundary management involves specialized actors for managing the interface between knowledge systems with clear lines of responsibility and accountability to opposite sides of the boundary; and use of ‘boundary objects’, i.e. palpable objects that all involved knowledge systems can directly understand in their own terms. Boundary objects focus communication, illustrate what actors refer to and thus enhance the mutual understanding of different viewpoints [17]. Three requirements are important for successful boundary management: (i) repeated and inclusive communication (to create access), (ii) mediation (to ascertain procedural and substantial fairness, adequate levels of relevance and scientific adequacy), and (iii) translation (to facilitate mutual understanding) [17–20].

Intercultural health is situated at the boundary of different medical knowledge systems, but literature on this topic is fragmentary [21]. Indeed, most of it concerns herbal medicine and local health beliefs and healing practices. Some efforts address barriers of access, patient choice [22–24], practitioner perspectives or attitudes to intercultural medical partnerships [25–27]. Several collaborative methods have been suggested to bridge the gap between biomedicine and traditional medicine in medically pluralistic settings, including workshop formats [28], comparative diagnoses as starting point to negotiate and understand meanings [29], and patient referrals to improve health services [8]. More complex approaches combine several of these elements into comprehensive research designs [30, 31]. Some of those methods were, for example, applied in Mexico [32–35], even though few projects have been conducted in true partnership with local communities from start to finish [21]. Literature does, however, mostly focus on medical content and few methodological studies assess such collaborative efforts empirically [36, 37]. Therefore, little is known about approaches to overcome barriers to successful implementation of intercultural health.

Specifically, we know of no study that (1) applies an integrated methodological design to foster partnership between biomedical and traditional practitioners in a collaboration that creates real value to all partners, and (2) comprehensively assesses intercultural processes and impacts that are triggered by that collaboration. These were important objectives in our case study in Guatemala, which was designed to investigate the role of a patient-centered boundary mechanism in creating access, building trust and fostering mutual learning between biomedical and traditional knowledge systems.

### Barriers to intercultural medical partnerships in Guatemala

Guatemala is a medium-sized country on the Central American isthmus. Of its population of 15 million people, 40% belong to 23 indigenous, mostly Mayan groups, each with their own language [38]. 52% of the population live in rural areas, and 51% are below the poverty line [39]. Access to local biomedical health services is usually good in the sense that the facilities are nearby and consultations are free. However, the cost of treatments may be prohibitive and the quality is often poor [40]. Referrals to higher-quality institutions entail more travel and higher costs. Communication between biomedical practitioners and the Mayan population is hampered by the linguistic diversity and differences in education, cultural expectations and explanatory models. These circumstances lead to mistrust towards biomedicine, as revealed in comments such as “not being attended” or “having to die” [41], and many patients are discouraged from accessing these services [22]. Furthermore, healers were explicitly targeted during the civil war (1960 – 1996), being regarded as local community leaders [42]. Finally, many doctors mistrust Mayan healers, whom they accuse of delaying the visits of patients to biomedical institutions, with the consequence that their conditions can no longer be treated effectively [43].

The history of inequality, racism and oppression of the Mayan population has been described as ‘structural violence’ [44–47]. As an outcome of the 1996 peace treaties, Mayan medicine is now officially recognised in the Guatemalan constitution, but this has almost no impact on medical practice [48]. For example, there are neither funding schemes nor formalized training opportunities [1]. Unsurprisingly, therefore, intercultural medical partnerships in Guatemala are perceived as unsuccessful [49], despite a few initiatives to improve the situation [50]. Overall, the Guatemalan context offers a case study to assess current limitations of intercultural health care and how these can be overcome through boundary management. It thus offers a model for the situation in many other developing countries.

## Research methodology

### Research design

The case study was conducted from January 2014 to May 2015 between ETH Zurich as science partner; the National Cancer Institute of Guatemala (INCAN) as a representative of biomedicine; and Councils of Elders of the Kaqchikel and the Q’eqchi’ Maya. Relations between ETH, Councils and INCAN had been initiated in previous collaborations in the fields of medical anthropology and ethnopharmacology, which is described elsewhere [51, 52]. On the Mayan side, the approach built upon existing trust relations that extended from Cirilo Perez Oxlaj (indigenous itinerant ambassador of Guatemala 2008-2012), through the Councils of Elders, to local healers and their patients.

Each partner had its own interests in participating (Table 1), and these are reflected in the mutually agreed objectives and fieldwork components:

1. Present biomedical conceptions of chronic, pervasive, non-infectious diseases to Mayan healers, operationalized through workshops given by INCAN to Mayan Councils and healers.
2. Identify and biomedically diagnose patients of healers suffering from such syndromes, operationalized through comparative diagnoses and patient referrals.
3. Document the patients health-seeking pathway, and Mayan conceptions of their illness and treatment, operationalized through interviews of patients, family members and healers.

**Table 1:** Interests that motivated the main involved partners to participate in the case study

Mayan Medicine/Councils	Science/ETH Zurich	Biomedical System/INCAN
Documentation and valorization of Mayan medicine	Gather scientific data on Mayan medicine	Learn about Mayan patients health-seeking pathways
Break cultural and historical barriers	Test a transdisciplinary research design to foster intercultural health	Understand Mayan patients high dropout rates

**Fig. 1:**
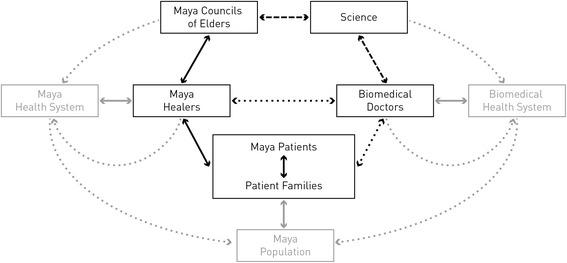
Diagram with actors and relations involved in the case study. *Left hand side:* Mayan knowledge system. *Right hand side:* Biomedical knowledge system. *Top:* study coordination and facilitation. *Center:* Medical systems. *Bottom:* Maya society. *Black:* Main actors and relations directly involved in the study. *Grey:* Contextual actors and relations influencing main actors. *Solid arrows:* relations that preexist the project. *Dashed arrows:* Relations established during a previous project [47, 48]. *Black dotted arrows:* relations established by the research design. *Grey dotted arrows:* Potential future indirect impacts of the study outcomes

Overall project coordination was in the hands of ETH, but implemented in a series of formal and informal facilitation and mediation workshops. These workshops were held throughout the process and were designed to discuss objectives and progress of the project, to clarify uncertainties and misunderstandings, to resolve potential conflicts, and to agree and plan next steps. INCAN took responsibility for all medical procedures and decisions. The Councils were responsible for the local coordination with healers and patients in their respective areas. The joint design and management thus guaranteed that the interests and concerns of all partners were addressed, and also helped ensure the scientific adequacy and relevance of the results. In these interactions, ETH researchers acted as external facilitators, neutral to the values and collective narratives of Mayan and biomedical systems in Guatemala. Jointly with the Councils of Elders, they legitimized the process, acted as intermediary between patients, biomedical and traditional practitioners, and mediated in case of misunderstandings, fears or conflicts. The network of actors and relations is summarized in Fig. 1.

ETH and the Councils jointly managed the interface of traditional and biomedical health systems. Accountabilities towards each side involved complying with the collaborative approach, agreed objectives, ethical constraints, and financial and administrative commitments. As boundary subjects [17], we focused upon clinical cases of patients suffering from chronic non-infectious diseases that are pervasive in the local population. These serve as palpable subjects to whom practitioners from both medical systems can relate to in the terms of their own knowledge and worldview. They thus enable interaction and coordination between biomedical and traditional practitioners, despite considerable differences in how these medical conditions are described and treated. In this way, the process becomes a boundary mechanism that facilitates intercultural medical partnerships – a space for communication and coordinated interaction that is connected to the realms of the two health systems, in which information can be translated from one system to another and conflicts can be mediated.

### Ethics, biomedical procedures and institutional platform

A Memorandum of Understanding (MoU) was signed between ETH Zurich and the Guatemalan Cancer League as the institutional body that runs INCAN. INCAN’s institutional review board (IRB) granted approval of the research design. INCAN committed itself as the partner solely responsible for all biomedical decisions and procedures. All diagnoses in the Kaqchikel area were conducted at INCAN. To reduce the patient’s travel and effort in the more remote Q’eqchi’ area, INCAN delegated some exams to regional departmental hospitals. Regardless of where the examinations were conducted, INCAN interpreted the results and decided on any follow-up examinations. Diagnoses conducted within this project did not differ in any regard from the medical procedures followed in standard medical services.

Collaboration with Councils and healers was based on a MoU between ETH and the National Council of Maya Elders. The councils accepted responsibility for local coordination, i.e. to follow up with healers and patients, to translate to/from indigenous languages, to accompany patients and to organize patient’s overland travel. In the Q’eqchi’ area, the councils also arranged for medical exams in local hospitals when requested by INCAN. These procedures between Councils, healers and patients were negotiated in workshops and contractually documented. Where needed, they were supported in these tasks by the project coordination team from ETH Zurich, and by local students.

Patients were fully informed by Councils in their native language and signed an informed consent sheet approved by INCAN’s IRB. In exchange, they received biomedical diagnoses, administrative, logistic and linguistic assistance, and coverage of all costs incurred by themselves and an accompanying person (transport, food, accommodation, reimbursement for the time investment).

### Data collection and data analysis

ETH developed three semi-structured interview guides for healers, patients, and family members. Each guideline contained approximately 80 questions covering personal information, the patient’s diagnosis and disease, health-seeking pathways, treatment strategies, social relations in the healing process, and follow up. The guidelines were validated in workshops with Councils and with two cases in the field. In the Kaqchikel area, interviews were conducted, recorded and transcribed by ETH researchers and local students in Spanish. In the Q’eqchi’ area, interviews were conducted and recorded by trained Council members in Q’eqchi’, then transcribed and translated into Spanish by a Q’eqchi’ linguist. Samples of any medicinal plants used were vouchered and identified at Universidad Del Valle’s Herbarium in Guatemala City (Herbarium code: UVAL). At several points in the process, traditional ceremonies were held to secure spiritual harmony and approval of the Mayan Councils of Elders. Thorough accounts of these methods and procedures are published elsewhere [51, 52]. After the fieldwork was concluded, an external researcher from Universidad Del Valle in Guatemala City evaluated experiences and perceptions of the project and its impact with eight key collaborators: four Mayan representatives (one healer and one Council member in each area), one surgical oncologist from INCAN, and three members of the project coordination team with personal access to all stakeholder groups and experience in observing subtle intercultural processes.

Based on literature knowledge and field experience, analytic categories were defined to understand the processes and impacts triggered by the project (e.g. illness of the patient, treatment strategies, health seeking pathway, access, trust, learning). Data was inductively sorted into these categories and entered into double entry matrices. Based on this thematic analysis, specific variables and indicators emerged and were quantified. To provide a comprehensive and balanced analysis, all variables relating to socio-cultural aspects of intercultural health in Guatemala, as well as project outcomes and impact were retained. Reflecting the scope and length of this methodological paper, medical data was summarized to merely give a general overview of the case study. Quotes and two example cases were selected to illustrate the process and the important findings. All names were changed to protect privacy.

## Case study results

### Partner engagement

INCAN held two kick-off workshops for Councils and healers in a culturally accessible style, mostly based on pictures of external symptoms, and verbal explanations of chronic, pervasive, non-infectious diseases. Healers asked questions and discussed Mayan terms for the diseases presented in the pictures. The workshops clarified the research focus and provided first opportunities for networking. After the workshops, 16 out of 25 healers showed an interest in the project. Six healers actively participated and suggested patients to participate in the research. The collaborative process was successfully completed with 35 patients. Two cases studies are described in detail in section 3.2. They exemplify the collaborative process and the effects it had on the interactions between healers, INCAN doctors and patients, and thus illustrate the sections 3.3, 3.4 and 4.

### Effects of intercultural medical collaboration

#### Don Manuel (Cog_Kaq_05): Avoiding an operation and accompanying his final days

In the first days of June 2014, Don Manuel (DM) visited Maya healer Nana Paulina (NP). DM was from an indigenous background and most of his family spoke little Spanish. He had been operated for colon cancer (right hemicolectomy) in a departmental hospital in 2013, and was subsequently referred to hospitals in Guatemala City for adjuvant chemotherapy. However, he never went there. His wife suffered from alcoholism and his daughter had become a prostitute, and, consequently, DM felt a duty to watch them 24 h a day. By this stage he was eating little, suffered from acute abdominal pain, and was pale, weak, and very thin. He had lost the will to live, but still hoped NP could help him.

NP worked to balance him emotionally and raise his confidence and self-esteem. In her diagnosis, she detected a malign spiritual disease (Kaqchikel: *Itzel Yab’il*) due to repeated disrespect towards a local deity, which caused the cancer to persist. She also concluded that he could no longer be healed since “*the light of his life had come to an end*” and the spirits “*had already prepared his path to the other life*” *(Cog_Kaq_05_h)*. Despite DM’s conviction that the cancer had been removed from him the previous year, he accepted her suggestion to visit INCAN for a diagnosis. On 23rd of June, blood tests and a tomography of chest and abdomen indicated that the colon cancer had recurred, spread to a kidney and the liver, and was in terminal stage. DM’s hemoglobin level was extremely low (3.4 g/dl, normal are 13-17 g/dl). Medical indications were emergency blood transfusions and palliative chemotherapy. Furthermore, an operation was scheduled in a week’s time to avoid his intestines from obstructing (ileostomy), and the family was informed that this would cost 15′000 GTQ (2′000 USD). DM remained at INCAN for 2 days, received transfusions until his hemoglobin was raised to 12.7 g/dl and then left the hospital.

NP talked more to the INCAN doctors, inquiring about the operation’s risks and consequences on DM’s immediate quality of life. On June 27th she visited DM and his family and explained in lay terms what had been discussed with the doctors. After some reflection, the family chose NP’s treatment rather than the biomedical one. NP gave DM two medications containing 7 plant species and one animal compound. One medication was intended to relieve his pain, and the other to produce diarrhea, thereby removing the intestinal obstruction. Fire ceremonies gave the family opportunity to reestablish harmony with the spirit world, while visits and talks with NP helped everyone prepare for DM’s imminent departure. According to NP, he departed calmly on July 11th, entering “*the other life*” without any problem.

**Fig. 2:**
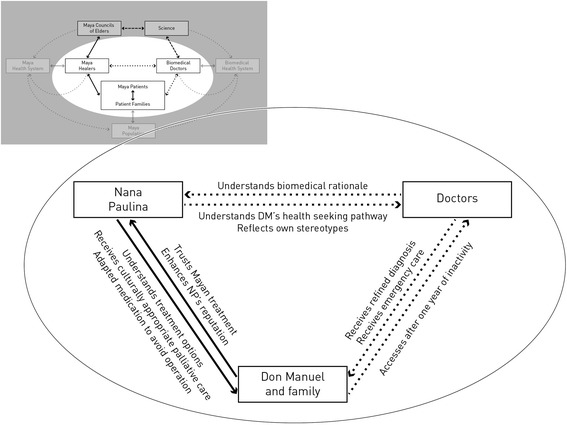
Effects that were stipulated by the collaboration between Nana Paulina and INCAN regarding Don Manuel

Later, NP reflected: “*What I told them was that this operation would not be a cure. I told them they should consider it well, to not regret or be disappointed afterwards, but that scientifically and spiritually, DM did not have much more time on earth. […] One of DM’s children asked me: You see, NP, if we had gone alone, we would have paid them, they would have operated him and he would have died anyway? I replied that what the doctors do is help the patient so that the intestines do not obstruct, but not to cure him. These are cultural misunderstandings. This was a very interesting experience, to learn how the doctors deal with situations, and how we deal with them. Since the doctor could not explain the situation to the family, he left this in my hands, so that I would explain it to them. So I took the role as spiritual guide, healer and psychologist. […] This is how I help my patients in final stages. I ease the pain, I involve the family and I prepare them to accept that one of their members is going to leave*” *(Cog_Kaq_05_h).* Figure 2 presents a summary of the interactions that evolved through this facilitated collaboration.

#### Doña Olga (Cog_Kaq_06): Healing an ‘incurable’ condition

On December 23rd, 2014, NP received a visit from Doña Olga’s (DO) husband and his brother. DM’s family had told them that NP was working in a project on cancer. Two months prior, DO, who was strongly rooted in a local church, experienced a strong abdominal pain. Since then she had been very sensitive to food, was sleeping badly, suffering from diarrhea, and could feel a swollen mass in her abdomen. She had already spent several thousand GTQ (several hundred USD) on visits to medical practitioners, pharmacies, local hospitals, and private clinics (7 total). Some of the people she visited had given her massage against colic, while others had prescribed painkillers and infusions against gastritis; but none of these treatments had provided anything more than temporary relief. She had also undergone tomography and ultrasound examinations. The last doctor suspected an advanced stage of cancer and referred her to specialists in the capital for an endoscopy, which the family however did not arrange. As DO recalled her reaction upon hearing this diagnosis from her husband: “*I asked him why he cried. I told him that as long as I am not dead, I needed help. I needed prayers, and I told to myself that in God’s name I did not have cancer. I had seen the power of God, the wonders that He has done in my live, I knew He would help me stand up again*” *(Cog_Kaq_06_p)*.

NP diagnosed the disease as sent by another person (Spanish: “*Mal enviado*”, a type of *Itzel Yab’il*), but still saw hope. She arranged DO’s examination at INCAN on December 26th. This revealed a severe urinary infection (urine with 18-20 leucocytes per microscopic field, normal is 0-1) and leukocytosis (22,540 leucocytes/ml, normal is <11′000). Some tumor markers (CA19-9, ACE y AFP) were negative, but ultrasound exams and tomography revealed a tumor that was strongly suggestive of pancreatic cancer. According to family members, the doctors estimated that she had only 3 weeks to live, and predicted that within days her pain would increase until no analgesic could help. Therefore, no treatment was prescribed.

As DO’s daughter recalled, upon arriving back home “*the house turned into a frenzy. Neighbors, other people, Catholics, Evangelicals, everybody came and left. For one and a half months they were here, came and left, prayed and did all sorts of things. And even now [June 2015] some are still coming” (Cog_Kaq_06_f).* NP conducted spiritual interventions according to Mayan tradition and prescribed a “*strong natural chemotherapy, analgesic and anti-inflammatory medication*” *(Cog_Kaq_06_h)* consisting of 3 medicinal plant species and two animal compounds. After 4 months, this treatment was changed to ingredients of moderate strength. Throughout this time (and in June 2015 still ongoing), the family visited NP every 15 days for more medicine, and independently proceeded with spiritual activities according to their catholic belief.

**Fig. 3:**
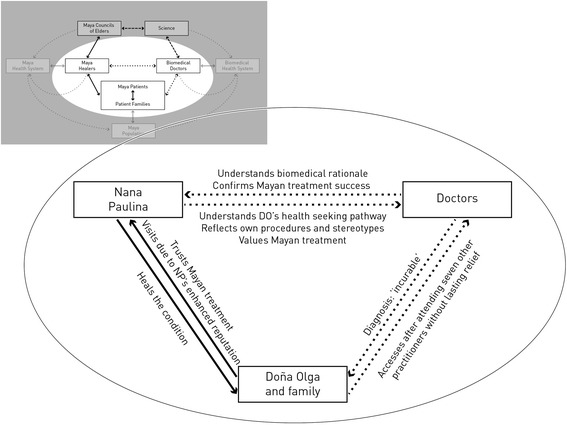
Effects that were stipulated by the collaboration between Nana Paulina and INCAN regarding Doña Olga

Apart from 1 day when the pain was particularly severe, DO showed slow but continuous improvement. Her daughter summarized DOs changes: “*She started feeling better, to eat, to stand up. Now she already does her daily duties, but still gets tired fast. […] She is still very thin, her face darkened somewhat, and she is losing her long, wild hair. She sometimes is a bit sad, but at other times she does not seem to worry. Spiritually, she is doing better and already walks in the streets […] Thank God, it is now already six months and here she is!” (Cog_Kaq_06_f)*. Furthermore, a tomography taken in June 2015 showed no sign of tumor. This led INCAN doctors to consider the possibility that her illness had been a severe pancreatitis, but even so they were surprised at her improvement. One reflected later: “*We cannot see anything anymore! Even though we could not do a biopsy, it was obvious that she had had something very serious. Her recovery is incredible!!” (Cog_Eval_3)*. Figure 3 presents a summary of the interactions that evolved in this facilitated collaboration.

### Process outcomes

These two examples emphasize the problems patients had to access adequate biomedical care before entering the case study. As Table 2 (top row) shows, this finding is frequent. Only four patients had visited a Mayan healer as their first choice. Twenty-eight patients had spent on average 7 years visiting one or several biomedical institutions, including health posts, pharmacies, private clinics, or local hospitals, before turning to a Mayan healer. Reasons for abandoning biomedical treatment included a lack of funds (14 patients), dislike of the proposed treatment (17 patients), and dissatisfaction with treatment outcomes (12 patients). For comparison, similar findings are reported from other Mayan patients in INCAN that chose to not attend a Mayan healer (Table 2, bottom row [43]. Nevertheless, 8 out of 15 Kaqchikel patients specifically contacted their healer since she worked in this project with INCAN (in Q’eqchi’ cases, this motivation was not mentioned). After completing biomedical diagnoses, all 35 patients continued Maya treatments, despite having the option to follow a biomedical treatment with doctors they now knew. Only in few cases was Mayan treatment supplemented with biomedical elements.

**Table 2:** Health-seeking pathways of patients in Guatemala

	Number of visited prior institutions		Time span between initial symptoms and arrival at Maya healer/specialized oncologist		Reasons for abandoning biomedical treatment/reasons for late attention of specialized oncologist
Patient group	Average	Range	Average	Range	
Patients of Maya healers interviewed in the case study	2	1–8	7.3 years	2 months–20 years	Lack of funds, disliking treatment experience, disliking treatment outcome
35 Maya patients at INCAN	3	1–7	3 years	3 months–12 years	Lack of funds, geographical access, lack of guidance, and language problems

Diabetes or elevated blood sugar levels were the most frequent medical conditions (15 patients). Non-malign or benign tissue affections (11 patients), or malign tissue affections (6 patients) were also common. Sixteen patients suffered from a wide range of other affections, including anemia, urinary problems and inflammatory problems. In most cases, Mayan healers accepted these biomedical diagnoses entirely (26 cases) or mostly (6 cases). Only in three cases did the two diagnoses diverge grossly. However, Mayan healers always interpreted the etiological origins of a disease in terms that were specific to the patient. Frequent categories were natural origins such as those resulting from a patient’s physiology, behavior or diet (19 times), social-emotional origins (12 times) and spiritual origins such as ‘sent’ diseases, spiritual misbehavior of the patient or birth signs (12 times).

In all 35 patients, Mayan healers prescribed medications consisting of 1-15 mostly herbal ingredients. In total, 80 species were applied, of which 71 (89%) could be collected and botanically identified. In addition, some animal compounds were used. Other prescriptions included ritual treatments (26 patients), behavioral or dietary changes (21 patients), and compresses, steam baths and massages (12 patients). Kaqchikel healers stated that biomedical diagnoses had influenced their treatment strategies for 9 out of 15 patients by complementing or confirming their own diagnosis. In contrast, the Q’eqchi’ treatments were – according to the healers’ interviews – not influenced by biomedical diagnoses.

### Impact evaluation

Table 3 presents the process evaluation. All interviewed collaborators agreed that the project had improved access among the various actors and that trust between the doctors and Mayan healers was strengthened (Table 3, Var. 1, 2). Factors that were specifically mentioned as improving trust were (1) better contact of doctors and healers, (2) the experience of having worked together with patients and thus being able to demonstrate the effectiveness of Mayan treatments, (3) the involvement of Councils of Elders, (4) adherence to Mayan procedures, and (5) reassurance that Mayan healers were not held responsible for patient’s advanced stage presentations at biomedical institutions.

**Table 3:** Project evaluations of eight key collaborators

		Pos.	Mixed	None	Neg.	NA
1	Access between patients, healers and biomedical doctors	8	0	0	0	0
2	Trust between Maya healers and biomedical doctors	7	0	0	0	1
3	Trust between Maya patients and Maya healers	1	3	2	0	2
4	Trust between Maya patients and biomedical doctors	2	2	2	1	1
5	Knowledge of *own* medical system	8	0	0	0	0
6	Knowledge of *other* medical system	8	0	0	0	0
7	Generalizability of process beyond individuals	1	4	0	1	2
8	Overall success as of expectations	4	3	0	0	1

Variables coded according to the project impact (except the variables “Generalizability” and “Overall Success”). Pos.: positive impact. Mixed: Mixed impact. None: No impact. Neg.: Negative impact. NA: Response does not cover variable or is unclear. E.g.: Seven collaborators saw a positive impact on the pre-project levels of trust between Maya healers and biomedical doctors

The responses regarding the trust of Mayan patients towards Mayan healers and biomedical doctors are mixed (Table 3, Var. 3, 4). In the Q’eqchi’ area, trust relations between healers and their patients were reported to be strong and unchanged throughout the project; however, intervention by the Council was needed to allay patients’ fears about visiting biomedical doctors. In the Kaqchikel area, one healer lost the trust of his patients by suggesting that they might participate in the project. Another healer reported no problems regarding the patients’ trust towards INCAN doctors; indeed, she even experienced a rise in her own prestige, with new patients visiting her to participate in the study. Reported reasons for these very different responses include cultural differences, more advanced cultural change in the Kaqchikel area and the manner in which Councils and healers explained the project and encouraged trust in its potential benefits.

All collaborators agreed that the project had taught them a lot regarding both their own and the other medical system (Table 3, Var. 5, 6). Frequently mentioned aspects were (1) learning about each other’s medical terms, procedures, diagnoses and treatments, (2) mutual understanding of the other’s disease classification systems, (3) characteristics of the health systems in general, (4) cross-fertilization among Maya healers, (5) understanding Maya patients’ health-seeking pathways, and (6) seeing beneficial effects of Maya treatments in some patients. It even influenced medical practice in both systems. One doctor stated: “*It opened my eyes to something we usually depreciate. […] It changed my way of looking at medicine in general. […] Especially the importance of spiritual healing concepts of Maya medicine, which I think is largely missing in biomedicine. […] I started to implement some of those with my private patients, obviously adapting it to occidental terms […]*” *(Cog_Eval_3)*. Similarly, one Kaqchikel healer stated: “*I could learn more about the illnesses of my patients and help them better. It helped me to see things in a new way, […] to develop new medicines, and include new ingredients” (Cog_Eval_5)*.

Proposals for up-scaling the process proved controversial (Table 3, Var. 7). Several collaborators pointed out that few people on either side were willing to get seriously involved in this process. Many healers were more concerned about the risks and problems than the opportunities and benefits, and only a few of INCAN’s doctors could see real value in the research. The Kaqchikel healers were reportedly criticized, sometimes severely, by their colleagues for ‘selling knowledge’, and one was even ejected from his local peer group. Healers also mentioned that some very deep knowledge could not be shared. On the other hand, there were also positive outcomes: the information on the health-seeking pathways of Mayan patients stimulated interest amongst doctors at INCAN and helped reduce misconceptions. On the Mayan side, the prestige gained by one healer could potentially serve as role model to spread the word more widely among her peers. Despite the weaknesses mentioned by some respondents, the project was therefore seen as mostly successful (Table 3, Var. 8).

## Discussion: Potential and limitations of this case study

### Intercultural partnerships discern barriers of access

Intercultural problems in access to biomedical care are well known. As exemplified in both case descriptions, this research demonstrates the challenges faced by Mayan patients; upon falling sick, most may visit several local biomedical institutions without success. In some cases they arrive in specialized care, though by then it may be too late to help them, or they may choose to change to Mayan healers. In either case, they often cannot afford specialized biomedical services, and/or are dissatisfied with its quality. Even the prospect of better access to biomedical services through the research project evoked contrasting responses, and, like Don Manuel and Doña Olga, all patients chose to remain in Mayan treatment after attaining biomedical diagnoses. Thus, the results partly confirm previous studies [22, 41, 44, 46, 49] that report shortcomings in the accessibility and quality of biomedical services, and reservations of the population to use them. However, our findings refute a belief that was widely held at INCAN before our study (and was also reported from other countries) that traditional healers are responsible for delaying patient’s visit to biomedical services [36, 43]. This exemplifies how evidence based research can address barriers to intercultural health care.

### Patient-centered collaboration builds trust and facilitates mutual learning

Previous studies have highlighted the relevance of trust for mutual learning and intercultural partnership [25, 36]. Despite the particularly challenging social and historical context of Guatemala in which previous efforts of intercultural medical collaboration have failed [1, 48, 49], empirical evidence demonstrates that the project was successful. The case study improved trust among practitioners, and there was ample evidence of mutual exchange and learning. Mayan healers accepted biomedical diagnoses as valid and complementary to their own, and, as in the case of Don Manuel and Doña Olga, even adapted their treatment strategies. Furthermore, they highly valued the opportunities to learn from both their peers and the doctors. As in the case of Don Manuel, Doctors were particularly impressed by the social realities behind Mayan health-seeking pathways, and the Mayan treatment outcomes they saw for example with Doña Olga. This triggered awareness for challenges of intercultural communication, reflection and a change of attitudes towards Mayan patients and healers. Finally, some collaborations on a personal level last beyond the conclusion of the case study and insights derived from the collaboration were implemented in treatments.

### Linking knowledge systems improves access and patient care

Intercultural partnership is currently an accepted aspiration in debates in global health, on the grounds of various benefits of traditional health systems [6–10]. This research was not designed to assess Mayan medicines effectiveness, even though treatment outcomes in some individual cases like Doña Olga were remarkable. It, however, suggests several benefits for the patients that specifically arose from linking traditional and biomedical knowledge systems. First, it provides an opportunity of facilitated access to biomedical services through involvement of traditional healers. As in the case of Don Manuel, this allows the patients to access biomedical institutions and to receive biomedical emergency care. Additionally, they also benefit from adapted and culturally pertinent traditional care. Finally, facilitation helped them to avoid mistakes and misunderstandings in situations that are challenging to them. Exemplified by Doña Olga, there was appreciation for such services among a segment of the Mayan population, who visited healers with the intention to join the project. This seems to depend on trust relations with healers and local context. Furthermore, the results demonstrate the reduction of doctor’s stereotypes towards the patients and their healers, and improvement of biomedical procedures due to enhanced self-reflection of doctors. Mayan health care improved due to refined biomedical diagnoses and adapted Mayan treatment strategies. Promoting these benefits might further stipulate interest in intercultural medical collaboration, even in areas or among groups that so far remained neutral or even skeptical.

### Multilateral boundary mechanisms facilitate intercultural medical partnership

These benefits were made possible by introducing several methodological innovations to the literature on intercultural collaboration. So far, research that is conducted in true partnership with local communities is generally lacking [21], and the few exceptions [37, 53] are restricted to bilateral settings that involve researchers and one group of local partners (patients, or traditional practitioners), but not biomedical practitioners. In contrast, our approach rests on a multilateral platform between 4 major groups of actors. All are actively involved in research design and execution of workshop techniques [28], comparative diagnoses [29], and patient referrals [8]. This was made possible since the research addresses interests of all partners in a coherent research design, and allowed to focus on patient as boundary subjects, whose diseases can be conceptualized from different viewpoints without losing their medical identity [17]. In this process, the role of the scientists was not only to observe and analyse, but also to facilitate and mediate. In this, they were supported by the Councils of Elders, who ensured fairness, relevance and legitimacy from the indigenous perspective. Thus, the project becomes a boundary mechanism, bridging biomedical and traditional knowledge systems with the objective to create access, to build trust and to foster learning on a path towards intercultural health. The case study demonstrates the potential of boundary mechanisms to foster collaboration across knowledge systems, a benefit that was evidenced in other fields [15, 18, 19] but seem transferable to intercultural medical partnerships.

### The art of designing boundary mechanisms for intercultural collaboration

Despite these successes, building trust and intercultural partnerships remains a significant challenge that can only be achieved through long-term collaboration [37]. Indeed, our case study built on previous relations among the main actors, which were extended through facilitated collaboration between healers, doctors and patients. Furthermore, double roles such as ETH’s responsibilities as facilitator and scientific coordinator can lead to conflicts of interest and put into question the legitimacy of the mediation, since it could be hypothesized that the facilitation is serving unilaterally scientific ends, rather than trying to impartially bridge different systems. The responsible research unit’s long-standing experience with collaborative research approaches at the science-practice interface, and previous joint project work among the partners mitigated such concerns in this case study. Nevertheless, an institutional partner with the sole mandate of securing neutral and unbiased facilitation would be preferable, especially in large-scale efforts. Finally, the successes were strongly dependent upon individual personalities, contextual variables, and the appearance of neglect or resistance from less open-minded representatives of both systems. In our view, a jointly designed project is essential to secure the legitimacy and interest of local collaborators, which in turn implies the adaptation of methods and objectives to each specific context. Consequently, implementation requires more than “methodological instruction manuals”: assessment of local background, a strategic and wise selection of key collaborators, a high degree of self-reflection among all partners, and strong skills in facilitation, negotiation and project management. Finally, it seems realistic to start with small-scale efforts which are gradually extended after having created successful examples and role models. Despite these challenges, the case study exemplifies the benefit of multilateral boundary mechanisms to foster intercultural medical partnership, even in very challenging contexts. It thus offers a model to improve health care in many medically pluralistic countries with a high share of indigenous populations.

## Conclusion: A new approach for intercultural partnership in medically pluralistic settings

This study is among very few research projects aiming to bridge the gap between biomedicine and traditional health systems in medically pluralistic developing countries in which parts of the society suffers from structural violence. We present a novel approach to foster intercultural health care in such contexts that goes beyond prior unilateral or bilateral efforts: a patient-centered, multilateral collaboration that includes representatives of biomedicine and traditional medicine, as well as scientists and indigenous authorities. The research design combines workshops and interviews, alongside comparative diagnoses and patient referrals. As one of the first research projects that involve local partners from start to finish, these methods are embedded in a transdisciplinary process in which all four groups are involved in defining objectives and procedures, conducting the research and, partially, analyzing and disseminating the data. Building on previously established relations of trust, science and indigenous authorities jointly take the role of facilitating communication, translating information and mediating conflicts across the boundary between health systems, thus ensuring the process is seen as valid, fair, and relevant by all sides.

We show that the design created access, build trust and bridged biomedical and Maya medical knowledge systems in Guatemala, a country with deep societal schisms in which previous efforts towards intercultural health have failed. It provided support for patients to access biomedical services, highlighted shortcomings of the current systems and pointed to the need for intercultural collaboration. It also created trust between practitioners of both systems, which shows some long-term effects. The impact on trust between patients and healers, and between patients and doctors respectively, shows more ambiguity. This highlights, how much these are dependent on individual healer personalities and local context and requires more research. By bridging knowledge systems, learning effects were observed among all participants, including reduction of stereotypes towards the patients and their healers, improvement of biomedical procedures due to enhanced self-reflection of doctors, improved Maya health care due to refined biomedical diagnoses and adapted Mayan treatment strategies. In a long-term perspective, potentials for integrating elements from both systems remain to be evaluated, but the surprising effects of Mayan treatments in individual cases are inspiring.

The design of boundary mechanisms for intercultural collaboration remains an art that requires careful consideration of local context and background, and wise selection of collaborators. Their general features seem, however, transferable and provide a promising avenue to foster intercultural health in developing countries. If scaled up and incorporated into wider audiences, boundary mechanisms can therefore contribute to reduce structural violence and improve health care in medically pluralistic settings.
